# Prognostic potential of neutrophil-to-lymphocyte ratio, platelet to lymphocyte ratio, and monocyte to lymphocyte ratio in acute myocardial infarction patients combined with chronic obstructive pulmonary disease

**DOI:** 10.3389/fcvm.2024.1401634

**Published:** 2024-07-12

**Authors:** Peizhu Dang, Feiyang Wang, Hang Yu

**Affiliations:** ^1^ Department of Cardiovascular Medicine, The First Affiliated Hospital of Xi’an Jiaotong University, Xi’an, China; ^2^ Department of Cardiovascular Surgery, The First Affiliated Hospital of Xi’an Jiaotong University, Xi’an, China

**Keywords:** acute myocardial infarction, chronic obstructive pulmonary disease, neutrophil-to-lymphocyte ratio, platelet to lymphocyte ratio, monocyte to lymphocyte ratio, prognosis

## Abstract

**Background:**

Inflammation is considered to play an important role in chronic obstructive pulmonary disease (COPD) and acute myocardial infarction (AMI), but the relationship between inflammation and poor prognosis in these patients has not yet been studied.

**Methods:**

We enrolled AMI patients combined with COPD and divided them into three groups according to the tertiles of neutrophil-to-lymphocyte ratio (NLR), platelet to lymphocyte ratio (PLR) and monocyte to lymphocyte ratio (MLR) respectively. Logistic regression analyses were used to identify risk factors for in-hospital all-cause death in these patients. Covariates were adjusted stepwise to determine the association between inflammatory markers and poor prognosis. Also, the receiver operating characteristic (ROC) curve was used to evaluate the greatest predictive indicator for all-cause death.

**Results:**

A total of 281 AMI patients combined with COPD were enrolled, of which 31 experienced in-hospital mortality. The risk of all-cause death was significantly higher among those with higher NLR. The highest tertile of NLR was significantly associated with an increased risk of all-cause death (all *P* < 0.05). This association remained significant after adjusting for confounding factors [Odds Ratio (OR): 10.571, 95% confidence interval (CI): 2.307–48.442, *P* = 0.002]. Moreover, compared to MLR and PLR, NLR had the highest predictive value for all-cause death [area under the curve (AUC): 0.764, 95% CI: 0.681–0.847].

**Conclusion:**

In AMI patients combined with COPD, elevated levels of inflammation were associated with increased all-cause mortality. Compared to other inflammatory indicators, NLR may provide a more superior predictive value.

## 1 Introduction

Chronic obstructive pulmonary disease (COPD) progressively inflames the respiratory system, affecting the airways, alveoli, and microvascular system. The incidence and mortality rates of COPD are increasing annually, causing approximately 3 million deaths worldwide. Despite improvement in treatment, the disease continues to impose substantial social and economic burdens (1, 2). Acute myocardial infarction (AMI) presents a significant challenge to global public health, with increasing incidence rates in our country (3). The pathological basis of AMI is the rupture of atherosclerotic plaques in the coronary arteries, resulting in the formation of complete, incomplete, or partial obstructive thrombi, which consequently cause a clinical syndrome of myocardial ischemia and necrosis (4).

Comorbidities are a crucial component of COPD, with cardiovascular diseases (CVD) being notably prevalent. Special attention is needed for AMI (5). Although the primary characteristic of COPD is localized pulmonary inflammation, previous studies have shown that it also leads to systemic spillover of elevated acute-phase proteins levels. This systemic inflammation can cause atherogenesis, promoting the development and expansion of arterial plaques. Recent researches highlight the indispensable role that chronic inflammation plays in the development of various diseases, including diabetes, CVD, and pulmonary diseases. The neutrophil to lymphocyte ratio (NLR), platelet to lymphocyte ratio (PLR) and monocyte to lymphocyte ratio (MLR) are recognized as crucial biomarkers of inflammation, reflecting the state of immunological equilibrium and prognosis (6, 7). Elevated NLR level is associated with a higher risk of all-cause and disease-specific mortality. Ball MK et al. found that high NLR in COPD patients is associated with an increased risk of AMI, stroke, and death (8). PLR is acknowledged as a marker of inflammation and atherosclerosis, with prognostic value for cardiovascular events (9). MLR has been recognized as a significant independent risk factor for all-cause and cardiac death (6).

Inflammation is a key factor in the pathogenesis of both AMI and COPD. Systemic inflammation and oxidative stress can promote the formation of atherosclerotic plaques in patients with COPD. Previous research has found that patients with both COPD and AMI have a higher long-term mortality rate (10), even after adjusting for recognized prognostic factors. However, no studies have yet explored whether this adverse prognosis is directly related to the inflammatory response.

Therefore, it is important to explore inflammation biomarkers for prognosis in AMI patients combined with COPD. However, there is limited research exploring the clinical characteristics of these patients, and the influence of inflammatory response on the prognosis of AMI patients with COPD lacks sufficient and reliable evidence. Therefore, this study aims to initiate a single-center study, retrospectively enrolled AMI patients combined with COPD from the First Affiliated Hospital of Xi'an Jiaotong University and analyzed the impact of NLR, PLR and MLR on short-term prognosis in these patients. This research tends to evaluate whether inflammatory markers can serve as predictors of adverse outcomes in these patients.

## 2 Methods

### 2.1 Population and groups

This is a cross-sectional study that included patients diagnosed with type I AMI combined with COPD at the First Affiliated Hospital of Xi'an Jiaotong University from January 2013 to January 2024. The study followed the principles of the Declaration of Helsinki and was approved by the Ethics Committee of the First Affiliated Hospital of Xi'an Jiaotong University (No: XJTU1AF2023LSK-515).

**Figure 1:**
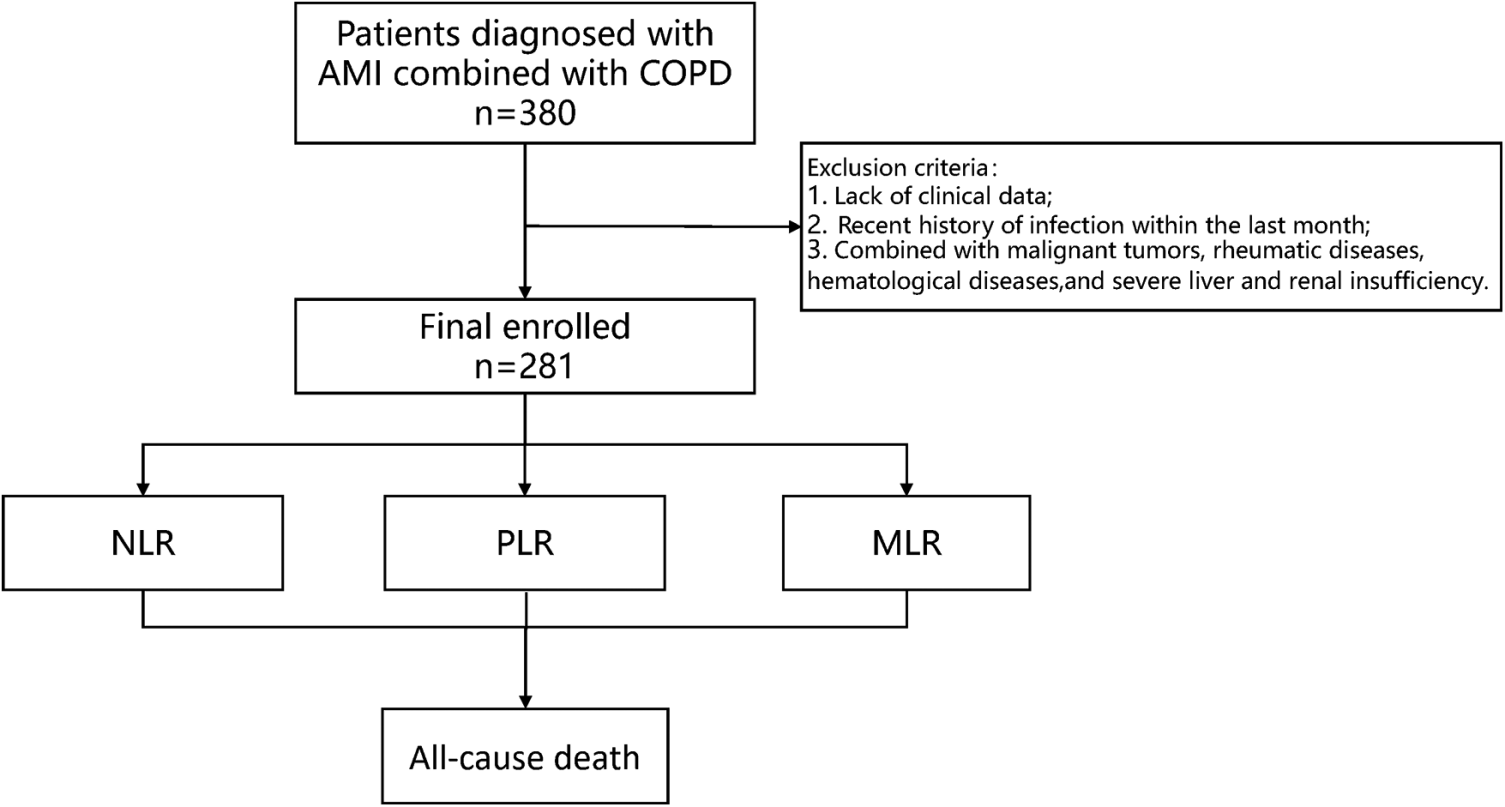
Study protocol. The flowchart of enrollment of study patients. AMI, acute myocardial infarction; COPD, chronic obstructive pulmonary disease.

The criteria for inclusion were as follow: (1) type I AMI patients combined with COPD; (2) 18–80 years. The criteria for exclusion were as follows: (1) incomplete clinical data; (2) recent history of infection within the last month; (3) combined with malignant tumors, rheumatic diseases, hematological diseases, and severe liver and renal insufficiency (Figure 1).

### 2.2 Data collection

On admission, all patients were assessed for demographic characteristics (age, gender), comorbidities (hypertension, diabetes, stroke, arrhythmia, smoking history), cardiac function and laboratory data. The laboratory data included complete blood count, liver and kidney functions, glycated hemoglobin, blood lipids. The primary endpoint of this study was the incidence of in-hospital all-cause death, which was obtained from the discharge records.

### 2.3 Measuring inflammation indicators based on blood cell counts

Blood cell counts were collected from patients at their admission to the hospital, including the total counts of neutrophil, lymphocyte, monocyte and platelet. Subsequently, composite inflammation ratios (including NLR, PLR and MLR) were calculated. The calculation of NLR is neutrophil count/lymphocyte count. The calculation of PLR is platelet count/lymphocyte count. The calculation of MLR is monocyte count/lymphocyte count.

### 2.4 Statistical analysis

Continuous variables were expressed as mean ± standard deviation for normally distribution data and quartiles for non-normally distribution data. Categorical variables were expressed as percentages. Descriptive statistics analyses were performed based on the levels of NLR, PLR and MLR, with subgroup differences were explored using Chi-square tests, Analysis of Variance, or Kruskal-Wallis *H*-test. Logistic regression analysis was performed to assess the association between inflammation ratios and all-cause death. And regression models were built, we performed three regression models: Model 1: unadjusted model; Model 2: adjusted for age, gender, and smoking, hypertension, diabetes, stroke, arrythmia; Model 3: adjusted for model 2, and creatinine (Cr), low-density lipoprotein (LDL), lactate dehydrogenase (LDH), creatine kinase (CK), CK-MB, aspartate aminotransferase (AST), alanine aminotransferase (ALT), and cardiac function. We used an alpha level of 0.05 for the inclusion of variables and 0.1 for the exclusion of variables, employing a backward stepwise method to build the regression model. The statistical analyses were performed by SPSS 27.0 (IBM Corp., NY). All analyses were two-sided and *P* < 0.05 was considered statistically significant.

## 3 Results

### 3.1 Clinical characteristics of study participants

Baseline data for all patients were shown in Table 1, including 281 AMI patients combined with COPD. Patients within the high NLR group had poorer cardiac function, and these patients were also likely to have higher levels of liver enzymes, and cardiac enzymes. Also, arrhythmia was more common in the high NLR group (all *P* < 0.05).

**Table 1:** Clinical characteristics of AMI patients combined with COPD.

Patients	NLR				PLR				MLR			
	Low (*n* = 94)	Middle (*n* = 93)	High (*n* = 94)	*P* vale	Low (*n* = 94)	Middle (*n* = 94)	High (*n* = 93)	*P* vale	Low (*n* = 94)	Middle (*n* = 94)	High (*n* = 93)	*P* vale
Age (years)	69.00 (65.00, 74.25)	68.00 (63.00, 74.00)	71.00 (66.75, 76.00)	0.080	68.50 (62.75, 73.00)	71.00 (65.00, 75.25)	71.00 (66.00, 76.00)	0.033	69.00 (63.00, 73.00)	71.00 (64.00, 75.00)	72.00 (66.00, 76.50)	0.014
Male (%)	79 (84.04)	80 (86.02)	81 (86.17)	0.899	87 (92.55)	74 (78.72)	79 (84.95)	0.027	17 (18.09)	10 (10.64)	14 (15.05)	0.347
Current smoking (%)	33 (35.11)	37 (39.78)	41 (43.62)	0.489	49 (52.13)	29 (30.85)	33 (35.48)	0.007	37 (39.36)	39 (41.49)	35 (37.63)	0.864
Killip III/IV (%)	9 (9.6)	12 (12.9)	25 (26.6)	0.004	10 (10.6)	12 (12.8)	24 (25.8)	0.010	9 (9.57)	14 (14.89)	23 (24.73)	0.018
EF < 50% (%)	14 (14.89)	16 (17.20)	33 (35.11)	0.001	18 (19.15)	18 (19.15)	27 (29.03)	0.174	13 (13.83)	18 (19.15)	32 (34.41)	0.002
Comorbidities (%)												
Hypertension	45 (47.87)	45 (48.39)	52 (55.32)	0.522	47 (50.00)	45 (47.87)	50 (53.76)	0.717	38 (40.43)	49 (52.13)	55 (59.14)	0.035
Diabetes	27 (28.72)	22 (23.66)	21 (22.34)	0.565	27 (28.72)	17 (18.09)	26 (27.96)	0.171	21 (22.34)	24 (25.53)	25 (26.88)	0.762
Stroke	22 (23.40)	17 (18.28)	14 (14.89)	0.324	17 (18.09)	15 (15.96)	21 (22.58)	0.498	16 (17.02)	21 (22.34)	16 (17.20)	0.572
Arrythmia	19 (20.21)	21 (22.58)	34 (36.17)	0.028	19 (20.21)	26 (27.66)	29 (31.18)	0.220	18 (19.15)	18 (19.15)	38 (40.86)	<0.001
Laboratory data												
Cr (umol/L)	69.00 (58.75, 79.00)	69.00 (57.50, 76.00)	69.50 (60.95, 94.25)	0.228	69.00 (63.00, 83.00)	69.00 (59.93, 79.00)	69.00 (56.50, 83.50)	0.615	67.00 (55.75, 78.25)	69.00 (60.68, 77.50)	73.00 (63.00, 93.00)	0.004
LDL (mmol/L)	1.95 (1.67, 2.59)	1.95 (1.55, 2.37)	1.95 (1.35, 2.42)	0.202	1.95 (1.71, 2.60)	1.95 (1.42, 2.35)	1.95 (1.47, 2.42)	0.107	1.95 (1.67, 2.52)	1.95 (1.54, 2.52)	1.95 (1.35, 2.22)	0.025
LDH (U/L)	256.00 (208.15, 328.25)	291.00 (231.50, 373.50)	362.00 (277.50, 633.25)	<0.001	291.00 (218.85, 393.00)	286.50 (237.75, 363.25)	313.00 (260.50, 463.00)	0.109	262.00 (218.55, 350.93)	287.00 (231.75, 356.25)	349.00 (278.00, 578.05)	<0.001
CK (U/L)	98.50 (65.75, 252.45)	196.00 (95.00,513.00)	341.50 (126.00, 1,085.58)	<0.001	196.00 (91.00, 601.80)	196.00 (84.50, 465.25)	196.00 (65.00, 675.50)	0.708	129.00 (66.00, 357.00)	192.00 (86.50, 424.20)	287.00 (134.50, 889.70)	<0.001
CK-MB (U/L)	17.00 (12.60, 38.25)	26.00 (15.00, 44.85)	38.60 (16.90, 101.50)	<0.001	26.00 (14.90, 59.10)	26.00 (13.00, 55.00)	24.00 (13.00, 71.90)	0.396	19.95 (12.85, 45.30)	26.00 (13.14, 44.00)	29.50 (20.99, 92.09)	0.001
ALT (U/L)	28.00 (20.00, 39.25)	28.00 (19.25, 34.00)	32.50 (24.75, 57.25)	<0.001	22.00 (28.00, 39.73)	28.00 (18.925, 40.25)	30.10 (21.50, 50.75)	0.159	28.00 (19.00, 41.93)	28.00 (19.15, 35.25)	31.00 (25.00, 51.80)	0.002
AST (U/L)	36.50 (23.50, 52.25)	42.00 (26.00, 74.00)	72.00 (33.50, 142.75)	<0.001	42.00 (28.00, 87.25)	42.00 (23.75, 76.50)	42.00 (26.50, 105.00)	0.365	37.00 (22.00, 68.61)	39.50 (24.75, 64.75)	71.00 (39.00, 136.50)	<0.001

AMI, acute myocardial infarction; COPD, chronic obstructive pulmonary disease; EF, ejection fraction; Cr, creatinine; LDL, low-density lipoprotein; LDH, lactate dehydrogenase; CK, creatine kinase; AST, aspartate aminotransferase; ALT, alanine aminotransferase.

The elderly and females were more likely to be in the high PLR group. Also, those with poor heart function, and arrhythmias were more likely to have higher MLR. These patients also tended to have higher levels of liver enzymes, and myocardial enzymes (all *P* < 0.05).

### 3.2 The association between composite inflammation ratios and all-cause death

**Figure 2:**
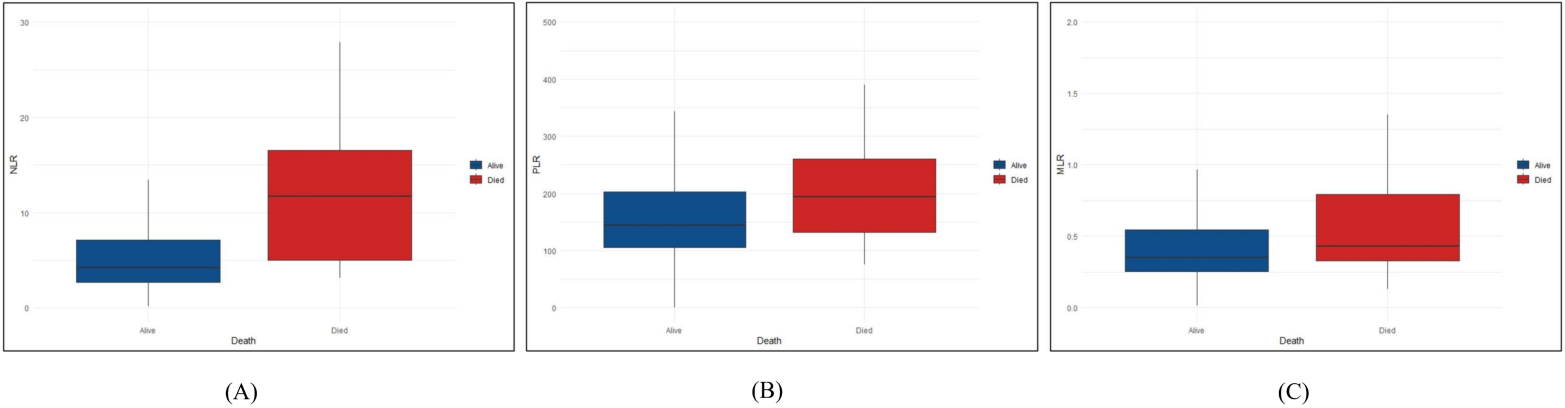
Differences in NLR, PLR and MLR between the died and alive groups. We described the distribution differences of NLR, PLR and MLR between died and alive groups. (**A**) The distribution differences of NLR between died and alive groups. (**B**) The distribution differences of PLR between died and alive groups. (**C**) The distribution differences of MLR between died and alive groups. NLR, neutrophil-to-lymphocyte ratio; PLR, platelet to lymphocyte ratio; MLR, monocyte to lymphocyte ratio.

A total of 31 (11.0%) patients died during their hospital stay, of which 9 died within 24 h of admission. Logistic regression analysis was used to assess the association between all-cause death and inflammation markers (Figure 2). The higher NLR was significantly associated with an increased risk of all-cause death [Odds Ratio (OR) = 11.653, 95% CI: 2.630–51.634, *P* = 0.001]. After adjusting for demographic characteristic (Model 2), this association remained significant (OR = 12.082, 95% CI: 2.689–54.290, *P* = 0.001). After further adjustment for biochemical indicators (Model 3), the positive correlation between NLR and all-cause death remained significant (OR = 10.571, 95% CI: 2.307–48.442, *P* = 0.002). Ultimately, the model we constructed through stepwise logistic regression analysis is: logit [P(Death)] = −7.690 + 0.060⋅Age—1.090 Hypertension + 0.001 AST + 1.768⋅NLR class1 + 2.358⋅NLR class2. However, this association was not found when PLR and MLR were considered as categorical variables (Tables 2–4).

**Table 2:** Logistic regression analysis of NLR in AMI patients combined with COPD.

NLR	Model 1		Model 2		Model 3	
	OR (95%CI)	*P* value	OR (95%CI)	*P* value	OR (95%CI)	*P* value
Quartile 1 (≤3.45)	Reference		Reference		Reference	
Quartile 2 (3.45–6.56)	5.542 (1.180, 26.031)	0.030	5.811 (1.225, 27.577)	0.027	5.860 (1.231, 27.912)	0.026
Quartile 3 (≥6.56)	11.653 (2.630, 51.634)	0.001	12.082 (2.689, 54.290)	0.001	10.571 (2.307, 48.442)	0.002

OR, odd ratio; CI, confidence interval. Other abbreviations as in Table 1.

**Table 3:** Logistic regression analysis of PLR in AMI patients combined with COPD.

PLR	Model 1		Model 2		Model 3	
	OR (95% CI)	*P* value	OR (95% CI)	*P* value	OR (95% CI)	*P* value
Quartile 1 (≤121.58)	Reference		Reference		Reference	
Quartile 2 (121.58–195.65)	2.359 (0.786, 7.077)	0.126	2.197 (0.724, 6.663)	0.164	1.898 (0.564, 6.383)	0.301
Quartile 3 (≥195.65)	3.423 (1.190, 9.849)	0.022	3.174 (1.084, 9.294)	0.035	2.739 (0.752, 9.977)	0.127

Abbreviations as in Tables 1, 2.

**Table 4:** Logistic regression analysis of MLR in AMI patients combined with COPD.

MLR	Model 1		Model 2		Model 3	
	OR (95% CI)	*P* value	OR (95% CI)	*P* value	OR (95% CI)	*P* value
Quartile 1 (≤0.29)	Reference		Reference		Reference	
Quartile 2 (0.29–0.48)	1.746 (0.608, 5.016)	0.301	1.863 (0.637, 5.450)	0.256	2.028 (0.658, 6.250)	0.218
Quartile 3 (≥0.48)	2.821 (1.043, 7.626)	0.041	3.029 (1.084, 8.459)	0.034	1.864 (0.580, 5.992)	0.296

Abbreviations as in Tables 1, 2.

### 3.3 The ROC curve between composite inflammation ratios and all-cause death

**Figure 3:**
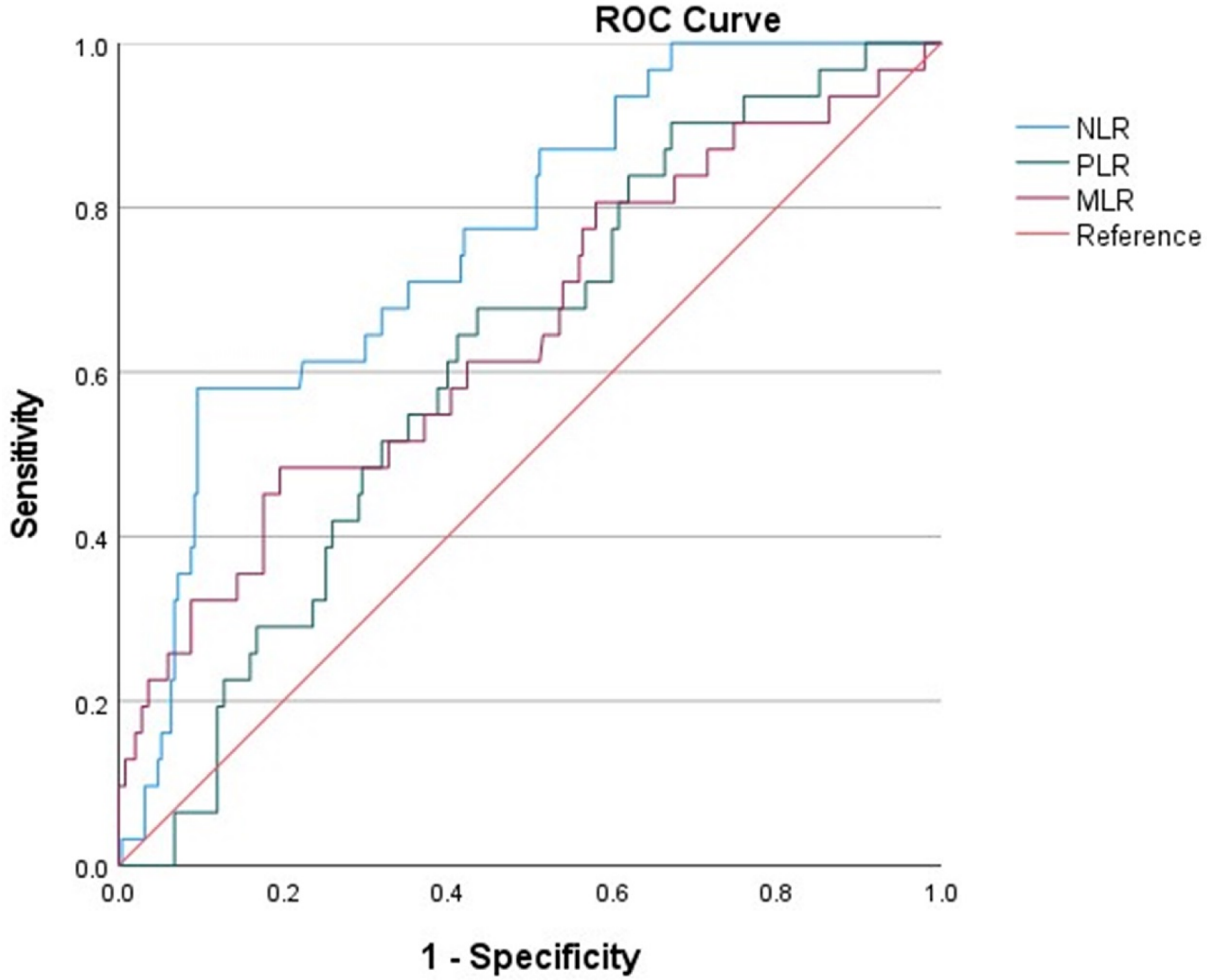
ROC curves for predicting all-cause death in all patients. ROC curves for predicting all-cause mortality plotted by the NLR, PLR and MLR in AMI patients combined with COPD. ROC, receiver operating characteristic; NLR, neutrophil-to-lymphocyte ratio; PLR, platelet to lymphocyte ratio; MLR, monocyte to lymphocyte ratio.

The AUC of NLR (0.764, 95% CI: 0.681–0.847) was significantly higher than that of other inflammation markers (Figure 3). The cut-off value was 11.434, with a specificity of 0.904, sensitivity of 0.581, Positive predictive value (PPV) of 0.4289, and Negative predictive value (NPV) of 0.946. The AUC of PLR and MLR were 0.619, 95% CI: 0.526–0.712 and 0.648, 95%CI: 0.537–0.758. The cut-off value of PLR was 163.478, with a specificity of 0.564, sensitivity of 0.677, PPV of 0.162, and NPV of 0.934. The cut-off value for MLR is 0.598, with a specificity of 0.804, sensitivity of 0.484, PPV of 0.234, and NPV of 0.926.

## 4 Discussion

In this study, a total of 281 AMI patients combined with COPD were included, among which 31 patients (11.0%) experienced in-hospital mortality. Patients with higher NLR values were found to experience a significantly increased rate of all-cause mortality compared to those with lower NLR, and the similar association was found in the MLR and PLR. After adjusting for potential confounding factors, NLR were identified as independent predictor of all-cause mortality. Notably, NLR showed superior prognostic value compared to PLR and MLR. To our knowledge, this is the first study to evaluate the predictive value of inflammatory markers for poor prognosis in AMI patients combined with COPD.

Previous studies have found the correlation between NLR, PLR and MLR with the severity of various diseases, including sepsis, pulmonary diseases and coronary artery disease (11). Our research has found that NLR was a significant predictor of poor prognosis in AMI patients combined with COPD. Although the prognostic value of inflammation for COPD patients with concomitant AMI has not been previously explored, previous studies have already identified NLR as predictor for AMI and COPD, respectively. NLR has been identified as a significant biomarker for both inflammation monitoring and prognosis in COPD patients. Shaghayegh Rahimirad et al. conducted a retrospective study on 315 COPD patients and found that the NLR values were higher in patients who died in the hospital compared to those who were discharged alive. Additionally, patients with higher NLR levels had a higher mortality rate than those with lower NLR levels (12). Paliogiannis et al. also found that NLR plays a significant role in predicting exacerbation and mortality in COPD patients (13). Ahmed Gouda El-Gazzar et al. found that NLR serves as a direct and valuable marker for predicting in-hospital mortality in COPD patients (14). A meta-analysis on the prognosis of COPD patients suggests that NLR may be an independent predictor of acute exacerbations in COPD patients. Furthermore, a high NLR may be associated with increased mortality in COPD patients, especially among Asians (15).

The potential mechanisms by which NLR is associated with poor prognosis in COPD patients may include: during the inflammatory process of COPD, neutrophils levels notably increase during severe acute exacerbations, particularly those induced by bacterial infections, though this increase is not exclusively linked to bacterial causes (16). Neutrophils release a substantial quantity of inflammatory mediators, contributing to the acute inflammatory response at the site of tissue injury (17). Neutrophils adhere to endothelial cells and cause lung tissue damage by discharging oxygen free radicals and proteolytic enzymes, ultimately leading to the destruction of alveoli and emphysema. Chronic immune stimulation has been observed to lead to an increase in lymphocyte levels. A low lymphocyte count is correlated with a poor prognosis in patients with acute diseases and chronic conditions, such as CVD and COPD. The reduction in lymphocytes, signaling a lower survival rate, is associated with factors characteristic of COPD, including being older and having a poor nutritional status (18).

In recent years, studies have observed that changes in NLR are a more reliable indicator of CVD than any other white blood cell subtype. Many studies have reported a significant association between elevated NLR levels and both cardiovascular mortality and all-cause mortality during hospital stay or long-term period in ST-elevation myocardial infarction (STEMI) patients (19). Zhenjun Ji et al. conducted a study involving 2,618 AMI patients and found that, compared to other common blood routine examinations (BRE), NLR was a better predictor of in-hospital mortality in AMI patients (20). Another study involving AMI patients found that high NLR levels were associated with a higher incidence of major adverse cardiovascular event (MACE) compared to lower NLR levels (21). Jingyu He et al. found that when AMI patients were stratified into tertiles based on NLR, patients in the T3 group had a 4.621 times higher risk of mortality compared to those in the T1 group (22). G-K Lee et al. found that NLR measured at admission, 24 h and 72 h after admission, and before discharge can all predict mortality in STEMI patients (23). This is consistent with our findings, the NLR was higher in the mortality group of AMI patients with COPD.

With inflammatory stimulation, the excessive production and release of neutrophils can promote the formation and progression of atherosclerosis. Also, neutrophils play an important role in the instability of atherosclerotic plaques, and they are the first subtype of white blood cells to infiltrate damaged myocardium, subsequently leaving the myocardial tissue after phagocytosing debris. During the onset and progression of AMI, the body experiences heightened inflammatory activation and increased cortisol levels. Elevated cortisol is known to induce apoptosis, resulting in lymphocyte reduction and an inversion of the CD4+/CD8+ T lymphocyte ratio. A significant decrease in CD4+ lymphocytes has been identified as a predictor of poor prognosis in AMI patients (24). Under the stress condition of myocardial infarction, the activation of the neuroendocrine system promotes lymphocyte apoptosis and inhibits the proliferation and differentiation of lymphocytes, leading to a reduction in peripheral blood lymphocyte count. Therefore, the decrease in lymphocyte count is closely related to the severity of myocardial infarction (25).

Comorbidities are recognized as a critical aspect of COPD, with CVD being one of the most common prevalent systemic manifestations that significantly impact these patients (26). Previous studies have demonstrated a strong association between COPD and CVD, particularly AMI. This association is caused not only by common risk factors but also by COPD-specific factors such as systemic inflammation and hypoxia, which contribute to the pathophysiological interplay between COPD and AMI. Although the exact mechanism by which COPD increases the risk of CVD remains unclear, previous studies have found that patients with COPD often exhibit elevated levels of systemic inflammatory biomarkers, such as C-reactive protein, IL-6, and fibrinogen, which can predict the risk of CVD in both the general population and patients with COPD (27). Maria-Elpida Christopoulou et al. thought that factors such as systemic inflammation, thrombosis, and oxidative stress associated with COPD make AMI closely related to COPD (28). The mechanisms underlying this association also include the unique physiological crosstalk between the lungs and the heart. This physiological interaction means that inflammation in the lungs can significantly impact the heart. Furthermore, systemic inflammation and hypoxia resulting from COPD have been identified as independent risk factors for AMI (29). CVD and COPD are both characterized by inflammation (30), and it's believed that systemic inflammatory response could significantly contribute to the pulmonary and systemic endothelial dysfunction (31). This highlights the importance of inflammatory markers in AMI patients with COPD. However, there are still few studies in this area.

Moreover, in patients with COPD, systemic inflammation may induce a procoagulant state, evidenced by elevated levels of tissue factor procoagulant activity and thrombin-antithrombin complexes. These changes lead to both pro-thrombotic and pro-inflammatory states (32). In CVD, the production of cytokines caused by activation of immune cells within atherosclerotic plaques, such as interferon-γ, tumor necrosis factor-α and acute-phase inflammatory proteins, including fibrinogen, amyloid and C-reactive protein, is observed (33). The mediators are observed in the inflammatory reactions in the bronchi of COPD patients. Previous studies have shown that chronic pulmonary inflammation can accelerate the progression of atherosclerotic plaques and that acute inflammatory episodes, such as acute exacerbations of COPD, can induce plaque rupture, potentially leading to cardiovascular events and worsening the prognosis (34). This is consistent with our findings that higher NLR levels were associated with increased mortality in AMI patients combined with COPD. NLR can systematically and accurately reflect the degree of inflammation and stress in these patients.

Notably, CVD is responsible for up to 1/3 of deaths in COPD patients (26). Laurien Goedemans et al. found the prognosis for COPD patients combined with AMI is notably poorer compared to those without COPD, including all-cause death and major adverse cardiac and cerebrovascular events (MACCE) during both short-term and long-term follow-up (5). By using the UK Myocardial Ischemia National Audit Program database, Kieran J Rothnie et al. found that patients with COPD had higher in-hospital and 180-day mortality rates compared to those without COPD (35). Previous studies have proposed that the increased mortality in these patients may be since they always present with atypical symptoms such as chest pain or dyspnea during acute phases. This can result in diagnostic and treatment delays (36), potentially leading to delayed reperfusion and an increase in infarct size, thereby worsening the prognosis (35). Our research considered that inflammatory responses play a significant role in this adverse prognosis.

Therefore, monitoring blood levels of systemic inflammatory biomarkers, particularly NLR, may provide an additional level of risk stratification for AMI patients combined with COPD. By analyzing the level of NLR, clinicians can gain valuable insights into the inflammatory status of these patients, which is crucial for identifying those at higher risk of adverse outcomes. Combining our findings with previous research, we propose that NLR is a valuable tool for accurately assessing the severity and prognosis of AMI patients with COPD. For high-risk patients identified through elevated NLR levels, several strategies can be implemented to decrease NLR and subsequently reduce mortality risk. These strategies include anti-inflammatory treatment (37), lifestyle improvements such as quitting smoking (38), maintaining a balanced diet, and engaging in regular physical activity (39), optimized management of comorbidities like hypertension and diabetes, early and aggressive intervention for patients with elevated NLR, and personalized treatment plans tailored to individual patient needs. By integrating these approaches, clinicians can more effectively manage inflammation and improve the overall prognosis for COPD patients with AMI, reducing their mortality risk.

Some limitations should be recognized. First, this is a single-center study with a small sample size. Second, due to the retrospective characteristic of the study, some clinical information could not be obtained. Third, the study was conducted in Northwest China, which may limit the generalizability of the findings to other populations. Future studies should include diverse geographical regions to validate and extend these findings. Finally, our findings need to be validated with external datasets to ensure the robustness and applicability of the results. Despite these limitations, this study can provide a basis for future research on the relationship between inflammation and adverse outcomes in AMI patients with COPD, promoting risk stratification and management for these patients.

## 5 Conclusion

In summary, NLR was independent risk factor for in-hospital death in AMI patients combined with COPD. Moreover, patients with higher NLR associated with a higher risk of in-hospital death compared to those with lower values. Clinicians should be more vigilant with patients who have higher inflammatory indicators, recognizing the heavier burden of adverse prognosis in such patients. Early identification and appropriate treatment are very important for the management of these patients.

## Data Availability

The original contributions presented in the study are included in the article/Supplementary Material, further inquiries can be directed to the corresponding authors.
